# Diversity in the Characteristics of *Klebsiella pneumoniae* ST101 of Human, Environmental, and Animal Origin

**DOI:** 10.3389/fmicb.2022.838207

**Published:** 2022-02-10

**Authors:** Sien De Koster, Juan Pablo Rodriguez Ruiz, Sahaya Glingston Rajakani, Christine Lammens, Youri Glupczynski, Herman Goossens, Basil Britto Xavier

**Affiliations:** Laboratory of Medical Microbiology, Vaccine and Infectious Disease Institute, University of Antwerp, Antwerp, Belgium

**Keywords:** *Klebsiella pneumoniae* ST101, livestock, community, hospital, environment, one health

## Abstract

**Background:**

*Klebsiella pneumoniae* ST101 is an emerging high-risk clone which exhibits extensive drug resistance. Bacterial strains residing in multiple hosts show unique signatures related to host adaptation. In this study, we assess the genetic relationship of *K. pneumoniae* ST101 isolated from hospital samples, the environment, community, and livestock using whole genome sequencing (WGS).

**Materials and Methods:**

We selected ten *K. pneumoniae* ST101 strains from hospitalized patients in Italy (*n* = 3) (2014) and Spain (*n* = 5) (2015–2016) as well as Belgian livestock animals (*n* = 2) (2017–2018). WGS was performed with 2 × 250 bp paired-end sequencing (Nextera XT) sample preparation kit and MiSeq (Illumina Inc.). Long-read sequencing (Pacbio Sequel I) was used to sequence the two livestock strains and three Italian hospital-associated strains. Furthermore, a public ST101 sequence collection of 586 strains (566 hospital-associated strains, 12 environmental strains, six strains from healthy individuals, one food-associated strain and one pig strain) was obtained. BacPipe and Kleborate were used to conduct genome analysis. ISFinder was used to find IS elements, and PHASTER was utilized to identify prophages. A phylogenetic tree was constructed to illustrate genetic relatedness.

**Results:**

Hospital-associated *K. pneumoniae* ST101 showed higher resistance scores than non-clinical isolates from healthy individuals, the environment, food and livestock (1.85 ± 0.72 in hospital-associated isolates vs. 1.14 ± 1.13 in non-clinical isolates, *p* < 0.01). Importantly, the lack of integrative conjugative elements ICE*Kp* bearing iron-scavenging yersiniabactin siderophores (*ybt*) in livestock-associated strains suggests a lower pathogenicity potential than hospital-associated strains. Mobile genetic elements (MGE) appear to be an important source of diversity in *K. pneumoniae* ST101 strains from different origins, with a highly stable genome and few recombination events outside the prophage-containing regions. Core genome MLST based analysis revealed a distinct genetic clustering between human and livestock-associated isolates.

**Conclusion:**

The study of *K. pneumoniae* ST101 hospital-associated and strains from healthy individuals and animals revealed a genetic diversity between these two groups, allowing us to identify the presence of yersiniabactin siderophores in hospital-associated isolates. Resistance and virulence levels in livestock-associated strains were considerably lower than hospital-associated strains, implying that the public health risk remains low. The introduction of an ICE*Kp* into animal strains, on the other hand, might pose a public threat over time.

## Introduction

*Klebsiella pneumoniae* is part of the Enterobacteriaceae family and is widely present in the gastrointestinal tract of humans and animals as well as in the environment. However, opportunistic, hypervirulent and multidrug-resistant (MDR) *K. pneumoniae* strains have emerged across the world (Martin and Bachman, 2018). *K. pneumoniae* causes a range of extraintestinal infections in humans, including pneumonia, urinary tract infections and bloodstream infections, usually in the context of opportunistic health-care-associated infections in vulnerable patient groups (Martin and Bachman, 2018). In the community, hypervirulent strains of *K. pneumoniae* can cause severe infections including pneumonia, pyogenic liver abscess, endophtalmitis, necrotizing fasciitis and meningitis in otherwise healthy persons (Wyres et al., 2020). In animals, *K. pneumoniae* is a common cause of bovine mastitis in dairy cattle (Holt et al., 2015), pneumonia in horses (Estell et al., 2016) and urinary tract infections in domestical animals (Marques et al., 2019) as well as septicemia, pneumonia and mastitis in pigs (Bidewell et al., 2018) and respiratory infections in broilers (Hamza et al., 2016).

The global success of the pathogen lies in its accessory genome, which plays an essential role in the emergence of high-risk isolates that are antibiotic-resistant and/or hypervirulent, and are associated with increased pathogenesis, invasive infections and fast adaptation to a specific niche or host (Hamza et al., 2016; Roe et al., 2019).

*Klebsiella pneumoniae* is part of the ESKAPE pathogens *(Enterococcus faecium, Staphylococcus aureus, Klebsiella pneumoniae, Acinetobacter baumannii, Pseudomonas aeruginosa, and Enterobacter* spp.) which accumulate AMR genes via horizontal gene transfer of plasmids and mobile genetic elements (MGE) (Wyres and Holt, 2018). The increasing occurrence of both virulent and MDR isolates (resulting from mutations in core genes as well as from the accumulation of horizontally acquired AMR genes) has led the World Health Organization to consider *K. pneumoniae* as a major global concern (Wyres et al., 2020). Healthcare-associated infections are usually caused by MDR clones with very limited or no treatment options. Especially, the global spread of carbapenemase-producing *K. pneumoniae* has become a reason for concern. *K. pneumoniae* sequence type (ST) 101 is one of the major high-risk clonal lineages of carbapanemase-producing isolates (*bla*_*OXA*–48_, *bla*_*KPC*_, *bla*_*NDM*_) and it has been associated with hospital-acquired infections worldwide (David et al., 2019), causing outbreaks in Algeria (Loucif et al., 2016), Czech Republic (Skalova et al., 2017), Greece (Avgoulea et al., 2018), Italy (Loconsole et al., 2020), Spain (Cubero et al., 2015), and Serbia (Palmieri et al., 2020). Colistin is a last-resort antibiotic for these infections (Fritzenwanker et al., 2018). However, colistin resistance has also emerged following its usage in difficult to treat infections caused by carbapenem- and multidrug-resistant *K. pneumoniae* including the ST101 high-risk clone, leaving almost no alternative treatment options and also leading to the dissemination of colistin resistance (Can et al., 2018).

Important virulence factors that contribute to pathogenicity include a capsule, lipopolysaccharides, siderophores, pili, iron uptake systems, efflux pumps and the type VI secretion system (Martin and Bachman, 2018). Virulence factors may be encoded by genes in the core genome (enterobactin locus *ent*, *fim*, and *mrk* loci encoding type 1 and type 3 fimbriae, K and O loci for capsular polysaccharide and LPS biosynthesis) and in the accessory genome (colibactin locus *clb*, salmochelin locus *iro*, aerobactin locus *iuc*, regulators of mucoid phenotypes *rmpA/A2* and the yersiniabactin locus *ybt*) (Wyres et al., 2020). Some of the latter genes are harbored on MGE including plasmids, transposons and integrative conjugative elements (ICEs). For example, the yersiniabactin (*ybt*)-encoding ICE*Kp* strongly influences the pathogenicity of *K. pneumoniae* strains. The *ybt* siderophore system is a key virulence factor that allows bacterial survival and replication in the host and is therefore significantly associated with pathogenesis and invasive infections (Lam et al., 2018). The *ybt* and ICE*Kp* structures are highly diverse and are sustained through dynamic horizontal gene transfer events (Lam et al., 2018).

In addition to reports of invasive infections, contamination of food animals or food products with MDR *K. pneumoniae* has been reported (Davis and Price, 2016; Wu et al., 2016; Projahn et al., 2019). Recently, the presence of NDM-1 carbapenemase-producing ST101 *K. pneumoniae* has been reported in chicken meat in Algeria (Chaalal et al., 2020). Likewise, other high-risk MDR clones, such as ST11 and ST258, have been detected in animals in China (Yang et al., 2019). Since *K. pneumoniae* is a colonizing opportunistic pathogen of both humans and animals and a common contaminant of retail meat, an increase in the future of the prevalence of MDR and/or of strains with enhanced virulence might constitute a potential threat for food safety as well as for animal and human health. On the other hand, a large fecal resistome study from slaughter pigs and broilers failed to identify carbapenemase genes suggesting that that these animals would apparently play a role of minor importance as reservoirs of clinical *K. pneumoniae* infections (Munk et al., 2018). To elucidate zoonoses, pathogen origin, virulence potential, genetic background and epidemiology of emerging infectious diseases, the investigations of bacteria from different origins is fundamental (Kirzinger and Stavrinides, 2012). In order to gain a better insight in the antibiotic resistance, virulence, and genetic relatedness between human (hospitalized patients and healthy individuals), animal (livestock-associated), food and environmental *K. pneumoniae* strains, we conducted antibiotic susceptibility testing and a WGS analysis on *K. pneumoniae* ST101 isolates from diverse origins to learn more about their diversity.

## Materials and Methods

### Strain Collection and Characterization

A total of 10 *K. pneumoniae* ST101 strains were collected within two point-prevalence surveillance studies of antibiotic-resistant *Enterobacteriaceae* in hospitals as well as in livestock farms. Strain (1101124) was isolated from feces of a broiler chicken in October 2017 and strain (1101433) was isolated from the feces of a weaned pig in February 2018 at two different livestock farms in Belgium within the framework of the i-4-1-Health study (Kluytmans-van den Bergh et al., 2019). Human strains were collected within the Resistance in Gram-Negative Organisms: Studying Intervention Strategies (RGNOSIS) study (ClinicalTrials.gov NCT02208154; EU-FP7, RGNOSIS). Three hospital-associated *K. pneumoniae* strains were collected from one single patient at one hospital in Italy in October 2014. The first two strains were recovered on the same day from an endotracheal aspirate (IT0132A) and from one rectal sample (IT0132R1). The third strain (IT0132R2) was collected 3 weeks later from a rectal sample. In addition, five strains were collected from screening specimens (throat and rectal samples) of five different patients at three different hospitals in Spain between April 2015 and August 2016 (BCR0495, BCR0504, BCR0133, FE1669, PS1684E). Further, all *K. pneumoniae* ST101 sequences originating from different sources [human including sequences from hospital-associated infections (*n* = 566) and healthy, asymptomatic carriers in the community (*n* = 6), animal (*n* = 1), food (*n* = 1) and the environment including river water (*n* = 1), hospital sewage (*n* = 7) and a surface at a NICU ward (*n* = 4)] available on NCBI and A Global Platform for Genomic Surveillance: Pathogenwatch on 27 September 2021 (*n* = 586) were added for comparison analysis (Supplementary Table 1). All sequences were added to the analysis to minimize bias because of selection.

### Antibiotic Susceptibility Testing

Minimum inhibitory concentrations (MIC) of ampicillin, cefotaxime, ceftriaxone, ceftazidime, ceftazidime-avibactam, imipenem, ciprofloxacin, norfloxacin, amikacin, gentamicin, tobramycin and trimethoprim-sulfamethoxazole were determined by a quantitative gradient diffusion method using ETEST^®^ (bioMérieux, Marcy l’Etoile, France) for ten study strains from humans (*n* = 8) and from livestock (*n* = 2). For colistin, MIC were determined using broth microdilution according to the ISO 20776-1 standard using 96-well polystyrene microplate (ref. 82.1582.001, Sarstedt, Nümbrecht, Germany). Results were interpreted according to EUCAST clinical breakpoints (v 10.0, 2020).

### DNA Extraction and Whole Genome Sequencing

All strains (*n* = 10) from this study were selected for short-read sequencing using the Illumina MiSeq platform (Illumina, San Diego, CA, United States). Briefly, for short-read sequencing, a single colony was inoculated in 4 mL Mueller-Hinton broth and incubated overnight at 37°C. Genomic DNA was extracted using the MasterPure Complete DNA and RNA Purification kit (Epicenter, United States) and purified with DNA Clean and Concentrator TM-10 Kit (Zymo Research, United States). DNA concentrations and quality were measured using a Qubit fluorometer (Thermo Fisher Scientific, Waltham, MA, United States). Libraries were prepared using the Nextera XT sample preparation kit followed by 2 × 250 bp paired end sequencing using MiSeq (Illumina Inc., United States).

Five *K. pneumoniae* ST101 strains from livestock animals (*n* = 2) and clinical isolates (*n* = 3) were selected for long-read sequencing on PacBio Sequel I (Pacific Biosciences, CA, United States). For long-read sequencing, high-molecular-weight DNA was isolated from fresh overnight cultures. Briefly, a single bacterial colony was inoculated in 10 mL Mueller Hinton broth and incubated overnight at 37°C under. DNA was extracted using the MagAttract HMW DNA kit (Qiagen, Hilden, Germany) according to the manufacturer’s protocol. DNA concentrations and quality were measured using NanoDrop (Thermo Fisher Scientific, Waltham, MA, United States) and Qubit fluorometer (Thermo Fisher Scientific, Waltham, MA, United States). Sequencing libraries were prepared using the SMRTbell Express Template Prep kit 2.0 (Pacific BioSciences) and whole-genome sequencing was performed on the PacBio Sequel I using the Sequel Sequencing kit 3.0 (Pacific BioSciences). The sequences were submitted in NCBI under BioProject PRJNA685961.

### Sequence Analysis and Genetic Characterization

Assembly of long-read sequencing data was performed with HGAP using default parameters, included in SMRT Link v8.0.0 (Pacific Biosciences). Short-read sequencing data was assembled using SPAdes (v3.13.0) (Bankevich et al., 2012). Assembly quality was assessed using Quast (v5.0.2) (Gurevich et al., 2013). Publicly available sequences of *K. pneumoniae* ST101 (*n* = 586) were downloaded from NCBI and from Pathogenwatch (Supplementary Table 1). Subsequent analysis was performed using BacPipe (v1.2.6) (Xavier et al., 2020), including the PubMLST database (Jolley et al., 2018), ResFinder (Bortolaia et al., 2020) and PlasmidFinder (Carattoli and Hasman, 2020) databases. The assembled short-read and long-read genomes were annotated using Prokka (v1.12) (Seemann, 2014) integrated in BacPipe. For the long-read sequences, insight in the accessory genome was obtained using web-based tools: PHAge Search Tool Enhanced Release (PHASTER) for identification of prophage regions (Arndt et al., 2016), IslandViewer using the IslandPath-DIMOB prediction method was used to identify genomic islands (Bertelli et al., 2017) and ISFinder predicted the presence of IS elements (Siguier et al., 2006). Recombinant whole genome sequences were identified using Gubbins (Genealogies Unbiased By recomBinations In Nucleotide Sequences) (Croucher et al., 2015).

Multiple alignment of genomes was done using Mauve (Darling et al., 2010). All sequenced isolates were screened for *in silico* K locus and O typing and presence of resistance and virulence determinants using Kleborate (Lam et al., 2021). Chromosomal insertion of ICE*Kp* structures was determined by the flanking direct 17 bp repeats “CCAGTCAGAGGAGCCAA” and ICE*Kp* variants were determined using Kleborate (Lam et al., 2018, 2021).

Statistical analysis was performed using a two-sample *t*-test assuming unequal variances.

Genome wide comparison was done using core-genome (cg) multilocus sequence typing (MLST). For cgMLST, a gene-by-gene approach was used by generating a study-specific scheme and analyzing cgMLST based allelic loci distance using ChewBBACA (Silva et al., 2018). Microreact was used to visualize allelic loci distances among isolates^1^.

## Results

### Antibiotic Susceptibility of *Klebsiella pneumoniae* ST101 From Humans and Animals

Higher MIC values for third generation cephalosporins, imipenem, fluoroquinolones and aminoglycoside antibiotics were consistently observed among hospital-associated strains compared to the animal strains (Table 1). Cephalosporin MIC were 24 to > 256 mg/L in hospital-associated strains compared to 0.032–0.094 mg/L in animal strains. Resistance to imipenem (4–6 mg/L) and to fluoroquinolones (ciprofloxacin MIC > 32 mg/L and norfloxacin MIC > 256 mg/L) in most hospital-associated strains was not observed in livestock strains. Resistance to aminoglycoside antibiotics in all but one hospital-associated strain was in contrast to MIC of 0.32–2 mg/L for these antibiotics in animals. Colistin resistance (MIC of 64 mg/L) was detected in one human and in one pig strain. In summary, the hospital-associated study strains showed an MDR phenotype (i.e., resistance to at least one agent in at least three antimicrobial categories) (Magiorakos et al., 2012) whereas animal strains were susceptible to most antibiotics.

**TABLE 1:** Minimum inhibitory concentrations (MIC) and interpretation for *K. pneumoniae* ST101 of human and animal origin determined by ETEST^®^ with the exception of colistin MIC which were determined using the broth microdilution method.

MIC (mg/L)										
	**Hospital-associated**								**Broiler**	**Pig**

**Antibiotic**	**IT0132A**	**IT0132R1**	**IT0132R2**	**BCR0495**	**BCR0504**	**BCR0133**	**FE1669**	**PS1684E**	**1101124**	**1101433**
Ampicillin	>256 (R)	>256 (R)	>256 (R)	>256 (R)	>256 (R)	>256 (R)	>256 (R)	>256 (R)	>256 (R)	>256 (R)
Cefotaxime	>32 (R)	>32 (R)	>32 (R)	>32 (R)	>32 (R)	>32 (R)	>32 (R)	>32 (R)	0.032 (S)	0.032 (S)
Cetriaxone	>256 (R)	>256 (R)	>256 (R)	>256 (R)	>256 (R)	>256 (R)	>256 (R)	>256 (R)	0.032 (S)	0.032 (S)
Ceftazidime	>256 (R)	>256 (R)	>256 (R)	32 (R)	24 (R)	32 (R)	>256 (R)	96 (R)	0.094 (S)	0.094 (S)
Ceftazidime-avibactam	1 (S)	1 (S)	1 (S)	0.5 (S)	0.5 (S)	0.5 (S)	>256 (R)	0.032 (S)	0.094 (S)	0.094 (S)
Imipenem	6 (R)	4 (I)	6 (R)	>32 (R)	6 (R)	6 (R)	>32 (R)	0.25 (S)	0.19 (S)	0.125 (S)
Ciprofloxacin	>32 (R)	>32 (R)	>32 (R)	>32 (R)	>32 (R)	>32 (R)	>32 (R)	>32 (R)	0.016 (S)	0.023 (S)
Norfloxacin	>256 (R)	>256 (R)	>256 (R)	>256 (R)	>256 (R)	>256 (R)	>256 (R)	>256 (R)	0.125 (S)	0.125 (S)
Amikacin	24 (R)	24 (R)	24 (R)	12 (R)	6 (S)	6 (S)	8 (S)	1.5 (S)	1.5 (S)	2 (S)
Gentamicin	96 (R)	128 (R)	128 (R)	192 (R)	48 (R)	64 (R)	96 (R)	0.25 (S)	0.38 (S)	0.38 (S)
Tobramycin	32 (R)	32 (R)	32 (R)	24 (R)	8 (R)	12 (R)	32 (R)	0.25 (S)	0.38 (S)	0.38 (S)
Trimethoprim-sulfamethoxazole	2 (S)	2 (S)	2 (S)	>32 (R)	>32 (R)	>32 (R)	0.125 (S)	>32 (R)	0.5 (S)	0.75 (S)
Colistin	≤ 0.125 (S)	0.5 (S)	64 (R)	0.25 (S)	2 (S)	0.25 (S)	2 (S)	0.25 (S)	1 (S)	64 (R)

*R, resistant; S, sensitive; I, intermediate.*

### Resistome and Plasmidome Analysis and Typing of *Klebsiella pneumoniae* ST101

In the hospital-associated study strains, resistance to aminoglycosides (gentamicin, tobramycin, amikacin) was correlated with the presence of aminoglycoside acetyltransferases [*aac(3)-IIa* and *aac(6′)-Ib*]. Resistance to the third-generation cephalosporins (cefotaxime, ceftriaxone and ceftazidime) could be explained by the presence of the extended-spectrum beta-lactamase (ESBL) genes *bla*_*CTX*–*M*–15_ and imipenem resistance was linked to the carbapenemase gene *bla*_*OXA*–48_ or *bla*_*NDM*–1_. High-level resistance to fluoroquinolone (ciprofloxacin resistance > 32 mg/L and norfloxacin resistance > 256 mg/L) was related to triple mutations in quinolone resistance determining regions of *gyrA* and *parC* as amino acid changes S83Y and D87N/D87G in GyrA and S80I in ParC and these were detected exclusively in clinical isolates. One hospital-associated strain (IT0132R2) contained an IS1 family IS1D inserted at position –100 in the promotor region of *mgrB* gene in contrast to the colistin sensitive strains from the same patient. In the pig isolate (1101433), a deletion of guanine at nucleotide position 116th nt led to a frameshift in *mgrB*. Both strains showed colistin resistance with an MIC value of 64 mg/L.

**FIGURE 1:**
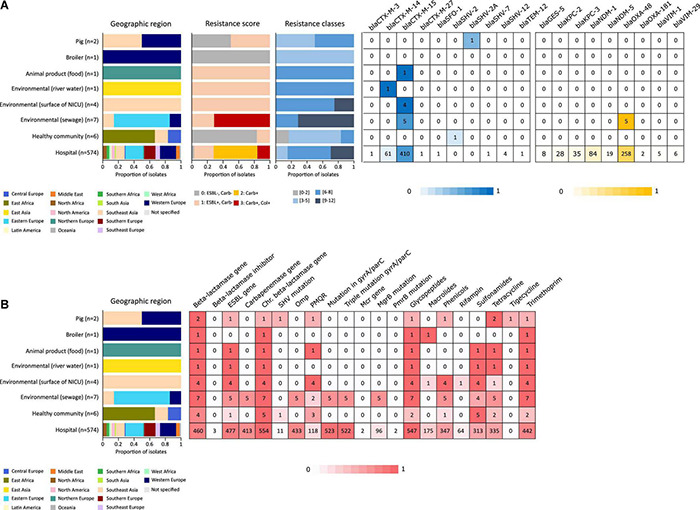
Resistance originating from ESBL- and carbapenemase production **(A)** and resistance to different antibiotic classes **(B)** detected in an international collection of hospital-associated, livestock-associated, healthy community and environmental *K. pneumoniae* ST101. Graphs and heatmaps show the proportion of isolates, numbers in the heatmaps indicate the number of genomes containing the resistance gene.

**FIGURE 2:**
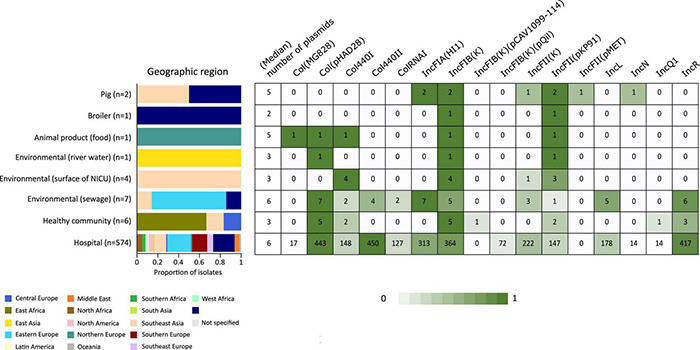
Heatmap of the most common plasmid origins of replication detected in *K. pneumoniae* ST101 based on screening against the PlasmidFinder database using BLASTn. Replicon markers shown are those with prevalence > 10% in one or more origin categories. Graphs and heatmaps show the proportion of isolates, numbers in the heatmaps indicate the number of genomes containing the resistance gene.

**FIGURE 3:**
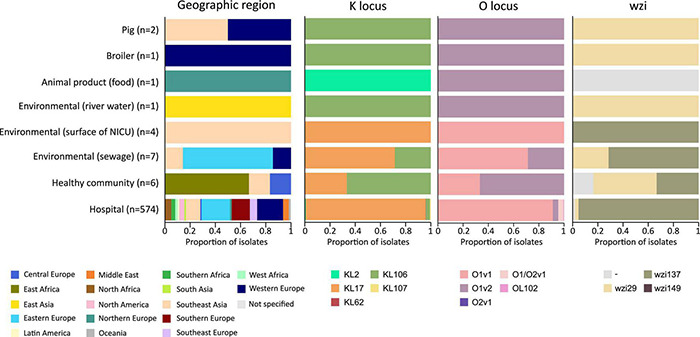
Typing of hospital-associated, livestock-associated, healthy community and environmental *K. pneumoniae* ST101.

In addition to the study strains, genotypic data was collected from 586 public sequences. Hospital-associated *K. pneumoniae* ST101 showed higher resistance scores compared to isolates from the environment, animals and healthy carriers in the community (1.85 ± 0.72 in hospital-associated isolates vs. 1.14 ± 1.13 in non-clinical isolates, *p* < 0.01) (Figure 1). Of all sequences originating from human clinical sources, 556 out of 574 (97%) showed ESBL- or carbapenemase production and 522 of 574 (91%) harbored triple mutations causing fluoroquinolone resistance (ParC S80I, GyrA S83Y and GyrA D87A/G/N). The most common ESBL gene was *bla*_*CTX*–*M*–15_ (420 out of 596 isolates, 70.4%) and the most common carbapenemase gene was *bla*_*OXA*–48_ (263 out of 596 isolates, 44.1%), though, *bla*_*CTX*–*M*–15_ or any carbapenemase gene were not detected in animal- nor in community-associated strains. The genetic context of *bla*_*KPC*_ carbapanemase genes has been further investigated. In this study, 63 out 574 (11%) of the sequences from hospital-associated isolates harbored a *bla*_*KPC*_ gene and were flanked by IS elements [ISKpn7/6, 59/63 (93.6%)], with only 4 (6.3%) flanked by transposon (tn2/tn3). The majority of the *bla*_*KPC*_ genes (*n* = 36, 64.8%) were carried on IncFII(K), with only 2 (3.2%) gene harboring on IncP6 and 5 strains (8%) containing IncFII(pKP91). The median number of plasmids was 6 in hospital-associated strains and strains from hospital sewage, 5 in the pig strains and food-associated strains and 2 or 3 in isolates from broiler, the environment (river water and surface at NICU) and the healthy individuals in the community (Figure 2). Plasmid replicon IncFIB(K) was detected in all categories and is known to be associated both with MDR and virulence plasmids (Wyres et al., 2020). The IncFIB(pQil) plasmid replicon is known to be associated with the pKPQil plasmid with resistance traits to *bla*_*KPC*–3_ and the *mer* operon (resistance to mercuric ions) (Navon-Venezia et al., 2017) and was detected exclusively in 72 clinical *K. pneumoniae* ST101 isolates (12.5%). Small plasmids (Col) were commonly detected in hospital-associated strains as well as IncR type plasmids (*n* = 417; 72.4%). Based on long-read sequencing data, Col-type plasmids did not harbor any resistance genes. The *bla*_*OXA*–48_ gene was carried on IncL plasmids which did not carry other resistance genes. However, various other Inc-type plasmids (such as IncFIB(K), IncFII(K), IncN and IncR) often carried a variety of resistance genes (up to 14 resistance genes) on one plasmid. Five distinct capsular polysaccharide (K loci) and LPS (O antigen) biosynthesis loci were defined among the *K. pneumoniae* ST101 strains (Figure 3). Serotype O1v1 and KL17 was the most common among hospital-associated *K. pneumoniae* isolates while O1v2 and KL106 was predominant in animal-associated and community-associated strains. Four hospital-associated isolates and one food-associated isolate from the publicly available databases carried KL2 which is highly conserved in hypervirulent clones and is associated with community-acquired invasive disease and enhanced pathogenicity (Wyres et al., 2020).

### Comparative Genome Analysis of *Klebsiella pneumoniae* ST101 of Different Origin Reveals That the Mobile Genetic Elements Are an Important Source of Variation

**FIGURE 4:**
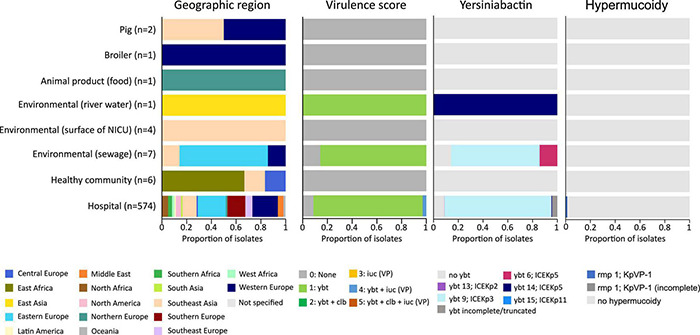
Virulence score, presence of yersiniabactin siderophore and hypermucoidy detected in an international collection of hospital-associated, livestock-associated, community-associated and environmental *K. pneumoniae* ST101.

The *K. pneumoniae* ST101 strains display a gene content associated with various horizontal gene transfer mechanisms such as plasmids, phages and MGE (e.g., ICE*Kp*). Intact yersiniabactin, a high-virulence determinant in *K. pneumoniae*, was present in a single genomic island in the majority of hospital-associated isolates (500 of 574; 87%) and 6 out of 7 isolates from hospital sewage (86%), however, yersiniabactin (*ybt* genes and ICE*Kp*) were absent in the six isolates from the healthy community, the two livestock isolates and in the isolate originating from food (Figure 4). This virulence factor was mobilized on the integrative conjugative element, ICE*Kp*, containing the *virB* operon of the type IV secretion system (T4SS) and the iron-scavenging siderophore yersiniabactin *ybt* locus. Five distinct *ybt* lineages were detected on four ICE*Kp* variants with *ybt9* on ICE*Kp*3 being the most common (495 (86%) of the hospital-associated isolates) (Figure 4 and Supplementary Figure 1). The MGE was detected in publicly available sequences collected during an infection as well as in sequences collected for screening. The ICE*Kp* corresponded to a 58–92 kb insertion integrated in an asparagine-tRNA in the chromosome. Based on long-read sequencing data (*n* = 12), the ICE*Kp* was inserted in the third asparagine-tRNA of the 4 or 5 asparagine-tRNA copies present in the chromosome (position 19,45,074–19,45,149 in reference sequence IT0132A) (Supplementary Figure 2). Insertion of ICE*Kp* occurred between a Na + /H + antiporter and HTH-type transcriptional regulator *argP* (*n* = 490; 98%), putative FMN/FAD exporter *yeeO* and endoribonuclease *pemK* (*n* = 4; 0.6%), between genes *mtfA* and *yjgH* (*n* = 6; 1.2%) or between *mtfA* and a Na + /H + antiporter (*n* = 1; 0.2%).

The ICE*Kp* was absent in the chromosomes of livestock-associated strains (*n* = 3). In the livestock-associated strains, the *virB* operon of the type IV secretion system was found on an IncN plasmid containing *bla*_*TEM*–1B_, *dfrA14* and *tetA* resistance genes and an IncFII(pMET) plasmid without resistance genes in the Belgian and in the Thai pig strains, respectively. The *virB* operon of the Belgian broiler strains was found on a IncP-like plasmid containing *aadA1*, *bla*_*TEM*–1B_, *Inu(G)* and *dfrB1* which was previously detected in an *E. coli* strain from pig caeca (accession number: CP039300.1). Of the 50 sequences from clinical strains in the public databases that lacked the *ybt* locus, five strains harbored the *virB* operon on a plasmid of which two contained the *dfrA14* gene for trimethoprim resistance.

**FIGURE 5:**
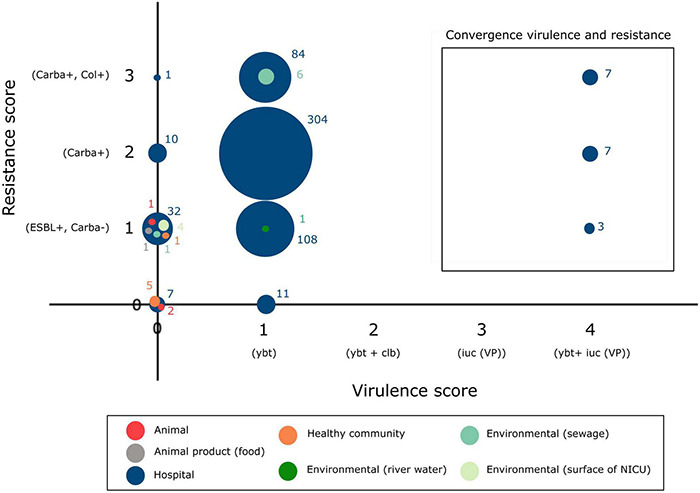
Convergence of antibiotic resistance and virulence in the *K. pneumoniae* ST101 population.

Besides the *ybt* locus, *rmpA/rmpA2* (hypermucoidy) and *iuc* (aerobactin) loci are other notable accessory virulence factors in *K. pneumoniae*. Seven publicly available sequences of hospital-associated strains harbored the *rmpA/rmpA2* genes indicating hypermucoidy. Convergence of resistance and virulence was detected in the sequences originating from 17 hospital-associated isolates from Italy (*n* = 8), Egypt (*n* = 5), Saudi Arabia (*n* = 2), Slovenia (*n* = 1) and Belgium (*n* = 1) (Figure 5). These sequences harbored the *ybt* and *iuc* loci in addition to ESBL-, carbapenemase genes and/or colistin resistance mutations/genes. Isolates from animals, animal products and the healthy community showed low virulence capacity (no *ybt*, *clb*, *iuc*) and no carbapenemase production.

**FIGURE 6:**
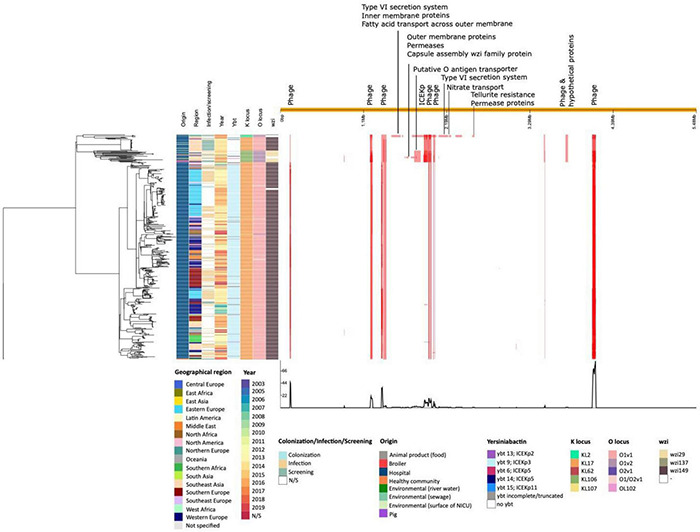
Phylogenetic analysis of an international collection of hospital-associated, animal-associated, community-associated (healthy individuals), food-associated, and environmental *K. pneumoniae* ST101. A maximum likelihood phylogeny was generated from whole genome alignment of 596 *K. pneumoniae* ST101 using the Gubbins algorithm. The right panel shows the pattern of predicted recombinations. Red bars show polymorphic sites suggesting horizontal sequence transfer. Each row relates to an isolate in the phylogeny and each column represents a base in the reference genome (IT0132A).

Based on long-read sequencing data, a total of 4.60–5.89% of the ST101 genomes was composed of genomic islands, and the percentage of prophage sequences was variable from 2.28 to 5.55% in both hospital-associated and in livestock-associated stains (Supplementary Figure 2). Prophage sequences did not contain any notable virulence factors. Distinct prophage content was detected in animal-associated and hospital-associated strains (Supplementary Figures 2, 3). A total of 468 polymorphic sites were identified across the *K. pneumoniae* ST101 genome (Figure 6). Genomic regions containing phage sequences, the ICE*Kp* region and regions harboring type VI secretion system, permease and outer membrane proteins were identified as recombinant. The latter were linked to diversity in K-and O locus types. The *K. pneumoniae* ST101 genome showed to be highly stable with few recombination events outside of these mentioned genomic regions (Figure 6).

### Analysis of the Sequence Data Revealed That Livestock-Associated Strains Were Genetically Distinct From Hospital-Associated Strains

**FIGURE 7:**
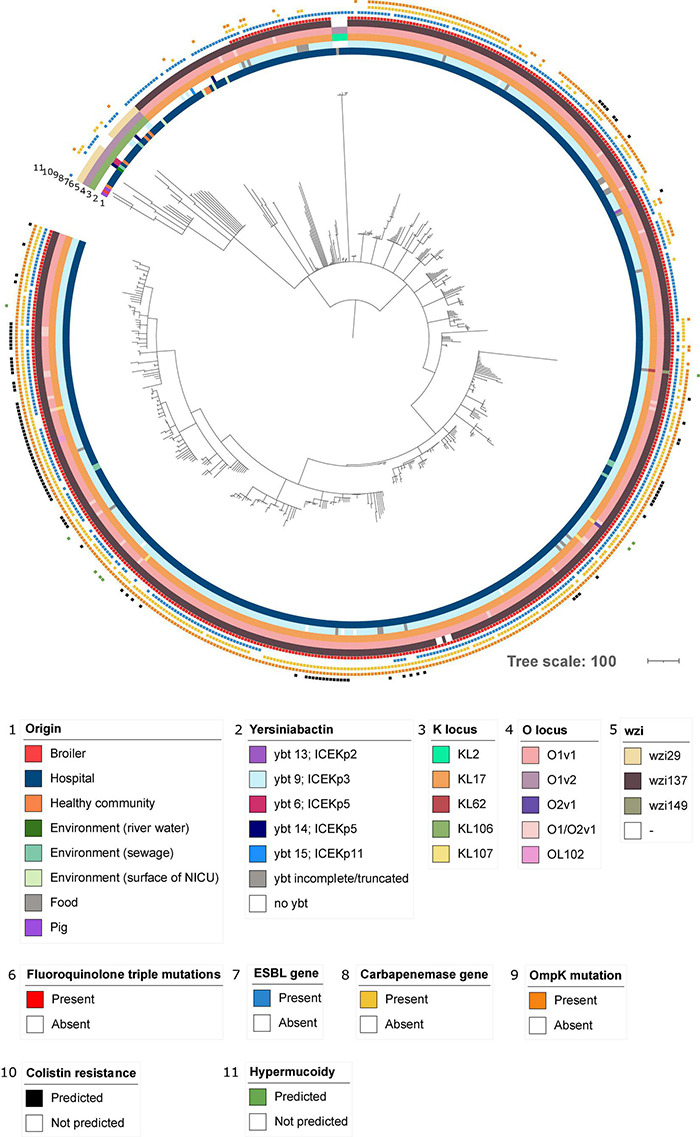
Phylogenetic analysis of hospital-associated, community-associated (healthy individuals), livestock-associated and environmental *K. pneumoniae* ST101. Figure was generated by iTOL using cgMLST profile data. Rings 1 and 2 picture the origin of the sequence and the presence of yersiniabactin. Rings 3, 4, and 5 indicate K locus, O locus and wzi type. The presence of resistance determinants including fluoroquinolone triple mutations, presence of an ESBL gene, presence of a carbapenemase gene, mutations in OmpK (OmpK35 and OmpK36) and mutations or genes predicted to be involved in colistin resistance are annotated in rings 6, 7, 8, 9, and 10. Predicted hypermucoidy based on the presence of *rmpA/rmpA2* is highlighted in ring 11.

To determine genetic relatedness, a study and strain-specific scheme was developed. A total of 4,427 loci were identified from ST101 isolates in the whole genome. 223 loci were deleted because they did not contribute to the core genome, leaving 4,202 loci for comparison among different ST101 clone origins (Figure 7). Overall, the gene-by-gene approach mirrored clustering based on K- and O-locus with the livestock-associated ST101 *K. pneumoniae* strains. The Belgian broiler strain (1101124) had 78 more and 164 fewer alleles than the strain KPSW02 from Thailand and Belgian pig (1101433) strains, respectively. The two pig strains had 86 core polymorphisms between them. The community-associated strain SB5560 from Madagascar was most similar to the livestock-derived strains, with a 95, 173, and 181 allelic distance to the pig strain from Thailand, Belgian broiler strain, and Belgian pig strain, respectively. The Swedish strain 08EU827 obtained from a feces sample of an ICU patient was the closest clinically relevant strain, with 211, 289, and 297 allelic variants in KPSW02, 1101124, and 1101433, respectively. The core genome of food strain F0025 was similar to Vietnamese, Swedish, and Canadian hospital-associated strains (less than 20 allelic differences) (Supplementary Table 2). Hospital-associated isolates did not cluster by region nor by the time of their isolation (Figure 6 and Supplementary Figure 4).

## Discussion

*K. pneumoniae* ST101 is an emerging high-risk opportunistic pathogen which has been reported mostly in hospital-outbreak settings in several countries (Cubero et al., 2015; Loucif et al., 2016; Skalova et al., 2017; Avgoulea et al., 2018; Loconsole et al., 2020; Palmieri et al., 2020). A broad collection of antibiotic resistance genes, including carbapenemase genes, is making ST101 highly adapted to the hospital environment (Roe et al., 2019). Indeed, a recent study showed that the nosocomial transmission of carbapenem-resistant *K. pneumoniae* substantially impacts the epidemiology of these clones in Europe (David et al., 2019). Although the detection of high-risk *K. pneumoniae* clones in animals remains scarce (Davis and Price, 2016; Yang et al., 2019; Chaalal et al., 2020), the spread of (resistant) bacteria between One-Health compartments exists (David et al., 2019). Hence, the occurrence of important nosocomial clones in animals may cause a reason for concern. In this study, we detected two *K. pneumoniae* ST101 in livestock animals [broiler chicken (*n* = 1) and weaned pig (*n* = 1)] in Belgium and eight nosocomial strains from Spanish and Italian hospitals. In addition, we analyzed an international collection of publicly available ST101 strains (*n* = 586). In this study, we provide insights into the genetic diversity of *K. pneumoniae* ST101 from the hospital (*n* = 574), healthy individuals in the community (*n* = 6), the environment (*n* = 12), food (*n* = 1) and livestock (*n* = 3).

MGE are an important source of variation between *K. pneumoniae* ST101 of different origin. The *K. pneumoniae* ST101 genome showed to be highly stable apart from the occurrence of a few recombination events outside of the MGE regions. The chromosomal insertion of self-transmissible ICE*Kp* elements in the clinical strains constitutes the main genomic difference between the *K. pneumoniae* chromosomes of animal and clinical origin in this study. This MGE was absent in the small number of available livestock-associated (*n* = 3), food (*n* = 1) and community-associated sequences (*n* = 6) of *K. pneumoniae* ST101 but provided many of the clinical strains (87%) with an advantage for the adaptation within the human host as it contains the virulence determinant yersiniabactin. This siderophore system scavenges iron from the host transport proteins and enhances the ability to survive and replicate within the host (Lawlor et al., 2007). In contrast to other siderophores, yersiniabactin also avoids the inflammatory response of the host (Bachman et al., 2011; Holden and Bachman, 2015). Yersiniabactin is, therefore, a key bacterial virulence factor and is significantly associated with invasive infections (Lam et al., 2018). We detected the ICE*Kp* element in hospital-associated strains from invasive infections as well as in commensal strains isolated from rectal or throat samples, most probably reflecting that the majority of the clinical sequences were deposited in the database in the context of difficult to treat (MDR) infections and/or linked to hospital outbreaks. This mobile cluster of genes showed genetic diversity between clinical strains as we detected four ICE*Kp* variants with different YbST which is probably indicative for long-term maintenance of ICE*Kp* in this lineage (Lam et al., 2018). The absence of yersiniabactin in some hospital-associated strains, livestock and community strains might be a consequence of the high-energy costs from the polyketide hybrid molecules and the ICE*Kp* cargo genes (Lam et al., 2018). Similarly, the absence of a fitness advantage of ICE*Kp* in the animal host might explain the absence of this ICE*Kp* in livestock strains. On the other hand, if the absence of the ICE*Kp* element in animal strains is due to the ecological barrier from the physical separation of bacterial populations in distinct host niches (Sheppard et al., 2018), high-pathogenicity and invasive strains could arise after the introduction of *ybt* in the animal strain background (Lam et al., 2018). The introduction of the ICE*Kp* in an asparagine-tRNA, an integration hotspot for genomic islands (Marcoleta et al., 2016), as observed in the hospital-associated strains might occur in the chromosome of animal strains over time. For the mobilization to recipient cells, the ICE*Kp* contains a *virB* operon. In livestock strains, the *virB* operon was detected on plasmids conferring antibiotic resistance. This type IV secretion system for genetic exchange may thus potentially act as an important contributor to genome plasticity and bacterial fitness via conjugation.

In the study collection of 596 sequences, hypervirulent clinical clones carrying a combination of core pathogenicity factors (K1 and K2 capsules; O1 and O2 LPS) with accessory virulence factors such as *rmpA/rmpA2* (*n* = 7) for hypermucoidy and *iuc* for aerobactin siderophore synthesis (*n* = 17) (Wyres et al., 2020) were detected. Convergence of resistance and virulence was not detected in isolates from animals, animal products and in healthy carriers in the community. These non-clinical isolates showed low virulence capacity and no carbapenemase production.

Of all hospital-associated strains, 97% showed ESBL-or carbapenemase production. When this is combined with resistance to fluoroquinolones, only limited treatment options remain (Fritzenwanker et al., 2018). Indeed, triple mutations causing fluoroquinolone resistance (ParC S80I, GyrA S83Y and GyrA D87A/G/N) were present in 91% of the hospital-associated strains and are known to be associated with a fitness advantage in high-risk MDR clones (Fuzi et al., 2017). Currently, the risk of acquiring MDR Enterobacteriaceae is linked to antibiotic selective pressure, contaminated drinking water and lack of hygiene (Nordmann et al., 2011). Colistin use in the Belgian pig farm was reflected by high-level colistin resistance in the pig strain, highlighting the importance of antibiotic selective pressure and the need to restrict antibiotic use in livestock. The public health risk posed by this opportunistic pathogen, taking into account its genotypic and phenotypic antibiotic resistance profile as well as the lack of critical high-virulence traits such as yersiniabactin in livestock-associated *K. pneumoniae* ST101 in this study, appears to be minor compared to hospital-associated strains.

However, there are some limitations to our research that must be addressed. The first limitation is the small number of livestock-associated *K. pneumoniae* ST101 strains available for analysis. Second, because the data was collected from a variety of sources, it does not capture precisely how prevalent *K. pneumoniae* ST101 is in livestock herds. The publicly available sequence data revealed large geographic and temporal variations in sampling (location, date of sample collection, and geographic regions), and it frequently failed to mention the clinical and epidemiological contexts in which the isolates were obtained and the sequences deposited. Presumably, most of the ST101 sequences were used to characterize MDR bacteria in a nosocomial or endemic environment, which is most likely why the prevalence of resistance and aggressiveness genes in our study was influenced. Third, the influence of the community on the introduction of these crucial nosocomial clones in livestock animals or vice versa is still unknown. As a result of these restrictions, raising WGS data from livestock-associated and community-associated strains is essential to detect the presence of these pathogens and their resistance and virulence genes. Our study, nevertheless, offers important information on *K. pneumoniae* ST101 resistance and virulence properties from a variety of origins, suggesting lower antibiotic resistance and the lack of high-virulence features such as yersiniabactin in livestock-associated, community-associated, and food-associated *K. pneumoniae* ST101 compared to hospital-associated strains. Future research should focus on the detection of clones in the community, in the hospital, and in livestock enclosures, employing a One-Health approach within a well-structured prospective study with representative sampling (geographic and temporal) in different sectors and settings.

## I-4-1-Health Study Group

Lieke van Alphen (Maastricht University Medical Center C, Maastricht, Netherlands), Nicole van den Braak (Avans University of Applied Sciences, Breda, Netherlands), Caroline Broucke (Agency for Care and Health, Brussels, Belgium), Anton Buiting (Elisabeth-TweeSteden Hospital, Tilburg, Netherlands), Liselotte Coorevits (Ghent University Hospital, Ghent, Belgium), Sara Dequeker (Agency for Care and Health, Brussels, Belgium and Sciensano, Brussels, Belgium), Jeroen Dewulf (Ghent University, Ghent, Belgium), Wouter Dhaeze (Agency for Care and Health, Brussels, Belgium), Bram Diederen (ZorgSaam Hospital, Terneuzen, Netherlands), Helen Ewalts (Regional Public Health Service Hart voor Brabant, Tilburg, Netherlands), Herman Goossens (University of Antwerp, Antwerpen, Belgium and Antwerp University Hospital, Antwerp, Belgium), Inge Gyssens (Hasselt University, Hasselt, Belgium), Casper den Heijer (Regional Public Health Service Zuid- Limburg, Heerlen, Netherlands), Christian Hoebe (Maastricht University Medical Center C, Maastricht, Netherlands and Regional Public Health Service Zuid-Limburg, Heerlen, the Netherlands), Casper Jamin (Maastricht University Medical Center C, Maastricht, Netherlands), Patricia Jansingh (Regional Public Health Service Limburg Noord, Venlo, Netherlands), Jan Kluytmans (Amphia Hospital, Breda, Netherlands and University Medical Center Utrecht, Utrecht University, Utrecht, Netherlands), Marjolein Kluytmans–van den Bergh (Amphia Hospital, Breda, Netherlands and University Medical Center Utrecht, Utrecht University, Utrecht, Netherlands), Stefanie van Koeveringe (Antwerp University Hospital, Antwerp, Belgium), Sien De Koster (University of Antwerp, Antwerp, Belgium), Christine Lammens (University of Antwerp, Antwerp, Belgium), Isabel Leroux-Roels (Ghent University Hospital, Ghent, Belgium), Hanna Masson (Agency for Care and Health, Brussel, Belgium), Ellen Nieuwkoop (Elisabeth-TweeSteden Hospital, Tilburg, Netherlands), Anita van Oosten (Admiraal de Ruyter Hospital, Goes, Netherlands), Natascha Perales Selva (Antwerp University Hospital, Antwerp, Belgium), Merel Postma (Ghent University, Ghent, Belgium), Stijn Raven (Regional Public Health Service West-Brabant, Breda, Netherlands), Veroniek Saegeman University Hospitals Leuven, Leuven, Belgium), Paul Savelkoul (Maastricht University Medical Center C, Maastricht, Netherlands), Annette Schuermans (University Hospitals Leuven, Leuven, Belgium), Nathalie Sleeckx (Experimental Poultry Centre, Geel, Belgium), Arjan Stegeman (Utrecht University, Utrecht, Netherlands), Tijs Tobias (Utrecht University, Utrecht, Netherlands), Paulien Tolsma (Regional Public Health Service Brabant Zuid-Oost, Eindhoven, Netherlands), Jacobien Veenemans (Admiraal de Ruyter Hospital, Goes, Netherlands), Dewi van der Vegt (PAMM Laboratory for pathology and medical microbiology, Veldhoven, Netherlands), Martine Verelst (University Hospitals Leuven, Leuven, Belgium), Carlo Verhulst (Amphia Hospital, Breda, Netherlands), Pascal De Waegemaeker (Ghent University Hospital, Ghent, Belgium), Veronica Weterings (Amphia Hospital, Breda, Netherlands), Clementine Wijkmans (Regional Public Health Service Hart voor Brabant, Tilburg, Netherlands), Patricia Willemse–Smits (Elkerliek Hospital, Helmond, Netherlands), Ina Willemsen (Amphia Hospital, Breda, Netherlands).

## Data Availability Statement

The datasets presented in this study can be found in online repositories. The names of the repository/repositories and accession number(s) can be found in the article/Supplementary Material.

## Ethics Statement

Ethical review and approval was not required for the animal study because the procedure to collect fresh fecal droppings is considered to cause no discomfort, and animals were neither handled nor sacrificed during the study (EC Directive 2010/63). Written informed consent was obtained from the owners for the participation of their animals in this study.

## Author Contributions

BX, YG, and HG: conceptualization. SD, JR, SR, and BX: data collection and writing. SD: writing—original draft preparation. SD, JR, SR, BX, and YG: writing—review and editing. CL: project administration. BX and HG: supervision. All authors have read and agreed to the published version of the manuscript.

## Conflict of Interest

The authors declare that the research was conducted in the absence of any commercial or financial relationships that could be construed as a potential conflict of interest.

## Publisher’s Note

All claims expressed in this article are solely those of the authors and do not necessarily represent those of their affiliated organizations, or those of the publisher, the editors and the reviewers. Any product that may be evaluated in this article, or claim that may be made by its manufacturer, is not guaranteed or endorsed by the publisher.

## Footnotes

1
https://microreact.org/project/j1fyqBYfCiKPZLa4qjotDe/ad1fbbb1
