# Supplementary material for: Diversity in the Characteristics of Klebsiella pneumoniae ST101 of Human, Environmental, and Animal Origin
Source: Front Microbiol. 2022 Feb 10;13:838207. doi: 10.3389/fmicb.2022.838207 (PMC8866942; doi:10.3389/fmicb.2022.838207)
Supplement: Supplementary file 3 [file Table_3.DOCX]

Supplementary Material

**Supplementary Figure 1.** ICE*Kp* structures detected in *Klebsiella pneumoniae* ST101. ICE*Kp* elements are flanked by direct 17 bp repeats ‘CCAGTCAGAGGAGCCAA’ (orange blocks).

**Supplementary Figure 2**. Alignment of *K. pneumoniae* ST101 chromosomes using long-read sequencing data and Mauve. Black dots represent IS elements, purple bars represent the ICE*Kp* elements, blue bars show genomic islands detected using the IslandViewer IslandPath-DIMOB prediction method. Red bars show incomplete phages, green bars show intact phages and orange bars indicate questionable phages as determined by the PHASTER software. Numbers 1-16 indicate the identified phage: 1) Phage_Salmon_SPN3UB_NC_019545, 2) Phage_Salmon_Fels_2_NC_010463, 3) Phage_Escher_phiV10_NC_007804, 4) Phage_Salmon_118970_sal3_NC_031940, 5) Phage_Entero_P88_NC_026014, 6) Phage_Cronob_ENT47670_NC_019927, 7) Phage_Entero_P4_C_001609, 8) Phage_Escher_RCS47_NC_042128, 9) Phage_Entero_c_1_NC_ 019706, 10) Phage_Salmon_SEN5_NC_028701, 11) Phage_Salmon_SEN34_NC_028699, 12) Phage_Salmon_vB_SosS_Oslo_NC_018279, 13) Phage_Escher_HK639_NC_016158, 14) Phage_Klebsi_phiKO2_NC_005857, 15) Phage_Pseudo_PS_1_NC_029066, 16) Phage_Entero_SfV_NC_003444.

**Supplementary Figure 3**. Chromosome length and length and percentage of genomic islands and prophage sequences in *K. pneumoniae* ST101 of livestock and hospital origin based on long-read sequencing data. Heatmap shows phage sequences (intact (green), incomplete (red) or questionable (orange)) detected in each genome.

**
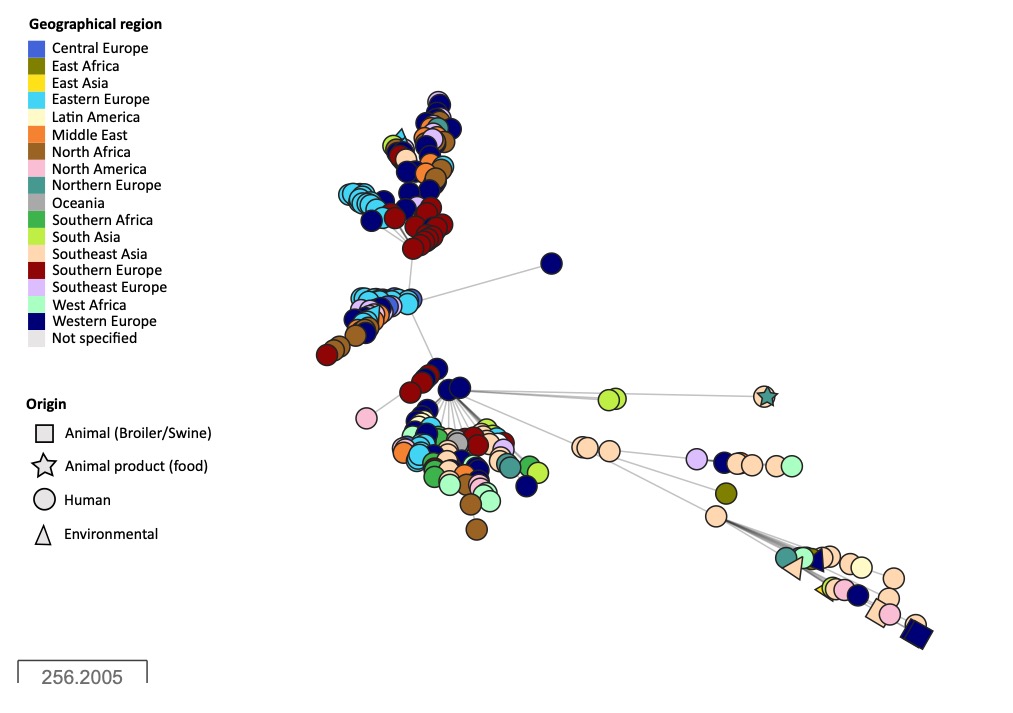
**

**Supplementary Figure 4**. Phylogenetic tree of an international collection of *K. pneumoniae* ST101 of human, animal and environmental origin based on cgMLST profile data. Interactive phylogeny is available at <https://microreact.org/project/j1fyqBYfCiKPZLa4qjotDe/ad1fbbb1>.
